# Supplementary material for: Extracts of Talaromyces purpureogenus Strains from Apis mellifera Bee Bread Inhibit the Growth of Paenibacillus spp. In Vitro
Source: Microorganisms. 2023 Aug 11;11(8):2067. doi: 10.3390/microorganisms11082067 (PMC10459140; doi:10.3390/microorganisms11082067)
Supplement: Supplementary file 1 [file microorganisms-11-02067-s001.zip › microorganisms-2541339-supplementary.pdf]

Supplementary materials to the research article:

Title:

"Extracts of Talaromyces purpureogenus strains from bee bread of *Apis mellifera* inhibit the growth of *Paenibacillus* spp. *in vitro*"

Authors:

Katerina Vocadlova, Tim Lüddecke, Maria A. Patras, Michael Marner, Christoph Hartwig, Karel Benes, Vladimir Matha, Petr Mraz, Till F. Schäberle, Andreas Vilcinskas

Content:

Table S1: Sequences used in the phylogenetic analysis.

Table S2: *Talaromyces* spp. isolates from bee bread of *A. mellifera* collected from different locations in South Bohemia.

Table S3: Cosine similarities and grouping of the *T. purpureogenus* strains extracts on different cultivation media.

Table S4: Minimum inhibitory concentration (µg/mL) of the crude methanolic extracts from *T. purpureogenus* strains.

Table S5: Minimum inhibitory concentration (µg/mL) of the reference antibiotics.

Figure S1-S5: Phylogenetic trees.

Figure S6: Locations of the apiaries, from which the bee bread samples were collected.

Figure S7-S9: MS chromatograms of the crude extracts of *T. purpureogenus* strains from different cultivation media.

Figure S10-S21: MS and MS/MS spectra of the active fractions and the precursor ions.

Table S1: Sequences used in the phylogenetic analysis with GenBank accession numbers. The new sequences are highlighted in yellow.

Strains	ITS	BenA	CaM	RBPII
Trichocoma paradoxa CBS 103.73	JN899399.1	JF417469.1	JF417506.1	JN121417.1
Talaromyces aurantiacus CBS 314.59	NR_103681.2	KF741917.1	KF741951.1	KC202951.1
Talaromyces zhenhaiensis ZH3 18	NR_177565.1	MZ054636.1	MZ054639.1	MZ054633.1
Talaromyces stipitatus CBS 375.48	NR_147424.1	KF741917.1	KF741951.1	KC202951.1
Talaromyces purpureogenus CBS 184.27	JX315665.1	JX315637.1	JX315658.1	JX315703.1
Talaromyces purpureogenus CBS 108923	JX965236.1	JX965343.1	JX965200.1	JX965303.1
Talaromyces purpureogenus CBS 113158	JX965235.1	JX965344.1	JX965201.1	JX965304.1
Talaromyces purpureogenus CBS 113161	JX965234.1	JX965345.1	JX965202.1	JX965307.1
Talaromyces purpureogenus CBS 286.36	NR_121529.1	JX315639.1	JX315655.1	JX315709.1
Talaromyces purpureogenus B11	OR192894	OR233621	OR327654	OR211402
Talaromyces purpureogenus B13	OR192895	OR233622	OR327655	OR211403
Talaromyces purpureogenus B18	OR192896	OR233623	OR327656	OR211404
Talaromyces purpureogenus B30	OR192897	OR233624	OR327657	OR211405
Talaromyces purpureogenus B49	OR192898	OR233625	OR327658	OR211406
Talaromyces purpureogenus B69	OR192899	OR233626	OR327659	OR211407
Talaromyces purpureogenus B195	OR192900	OR233627	OR327660	OR211408
Talaromyces purpureogenus CBS 122434	JX315663.1	JX315640.1	JX315659.1	JX315701.1
Talaromyces purpureogenus DTO189A1	JX315661.1	JX315638.1	JX315642.1	JX315699.1
Talaromyces panamensis CBS 128.89	JN899362.1	JX091386.1	KF741936.1	KM023284.1
Talaromyces flavus NRRL 2098	EU021596.1	EU021663.1	EU021694.1	EU021620.1
Talaromyces sparsus FS1 2	MT077182.1	MT083924.1	MT083925.1	MT083926.1
Talaromyces penicillioides 14061	MK837956.1	MK837940.1	MK837948.1	MK837964.1
Talaromyces haitouensis HR1 7	NR_177564.1	MZ054634.1	MZ054637.1	MZ054631.1
Talaromyces thailandensis CBS 133147	NR_147428.1	JX494294.1	KF741940.1	KM023307.1
Talaromyces aspriconidius CBS 141835	NR_170774.1	MN863343.1	MN863320.1	MN863332.1
Talaromyces francoae DTO 056D9	NR_154940.1	KX011489.1	KX011501.1	MN969188.1
Talaromyces aculeatus NRRL 2129	KF741995.1	KF741929.1	KF741975.1	MH793099.1
Talaromyces argentinensis NRRL 28750	NR_165525.1	MH792917.1	MH792981.1	MH793108.1
Talaromyces louisianensis NRRL 35823	NR_165526.1	MH792924.1	MH792988.1	MH793115.1
Talaromyces veerkampii CBS 500.78	NR_153228.1	KF741918.1	KF741961.1	KX961279.1
Talaromyces californicus NRRL 58168	NR_165527.1	MH792928.1	MH792992.1	MH793119.1
Talaromyces malicola NRRL 3724	NR_165531.1	MH909406.1	MH909459.1	MH909567.1
Talaromyces pratensis NRRL 62170	NR_165529.1	MH792948.1	MH793012.1	MH793139.1
Talaromyces domesticus NRRL 58121	NR_171608.1	MH792927.1	MH792991.1	MH793118.1
Talaromyces tumuli NRRL 62151	NR_165528.1	MH792944.1	MH793008.1	MH793135.1

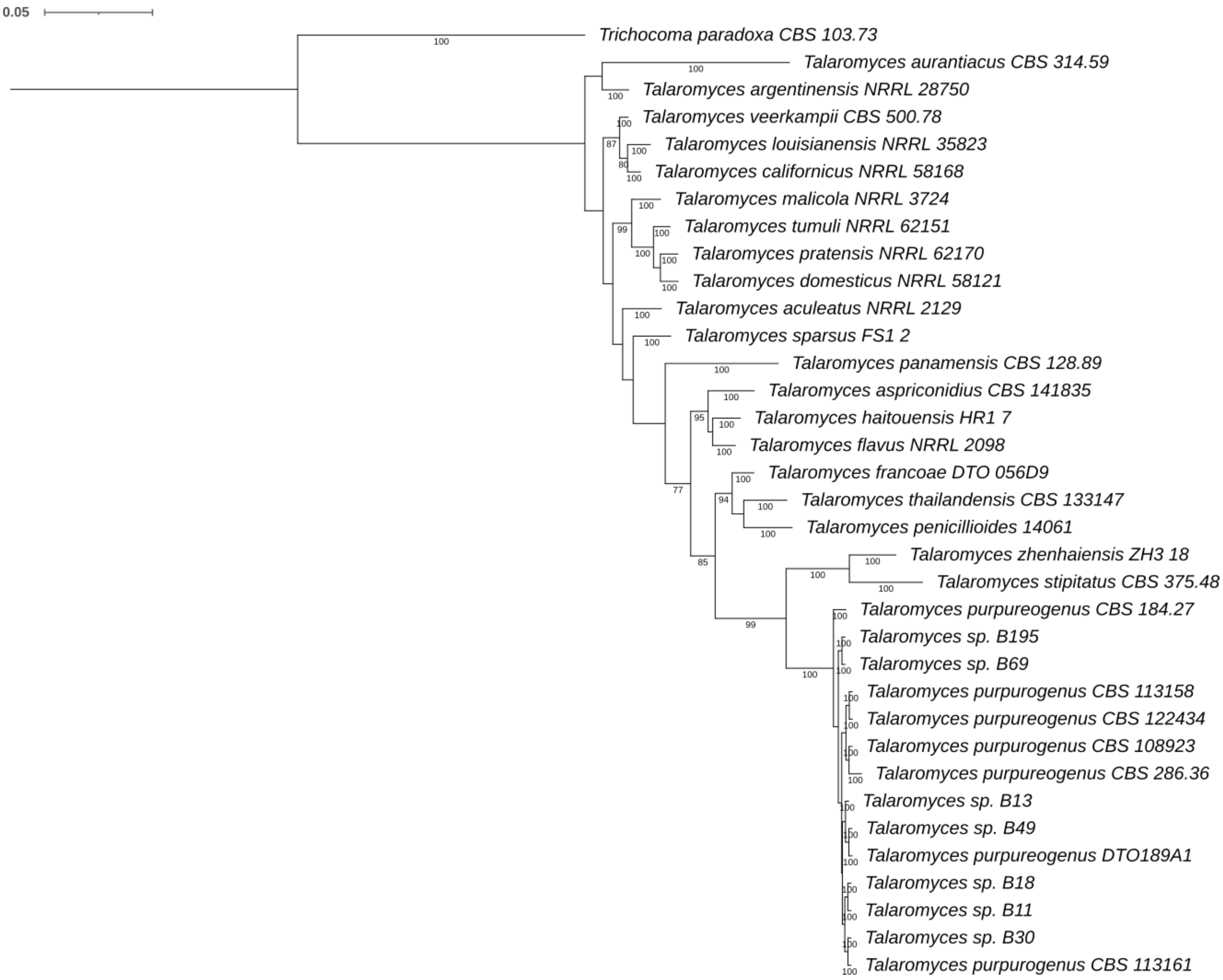

Figure S1: Phylogenetic tree based on the ITS, BenA, CaM and RBPII gene regions showing the relationship between the seven newly described strains of *T. purpureogenus* and other related members of the genus *Talaromyces.* Support in the nodes is represented by bootstrap values >70. *Trichocoma paradoxa* was used as an outgroup.

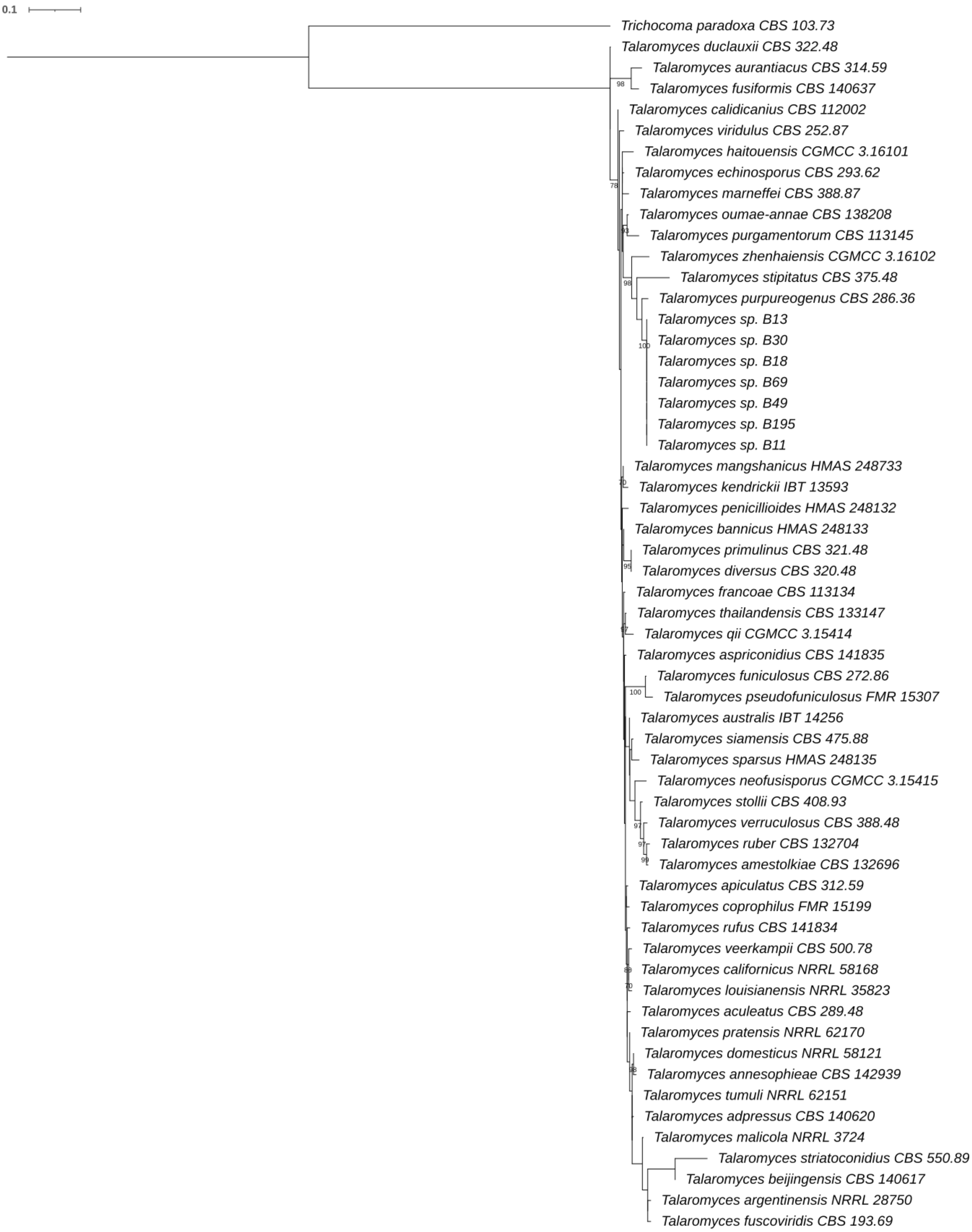

Figure S2: Maximum likelihood phylogenetic tree based on ITS region. 58tax Support in the nodes is represented by bootstrap values >70. *Trichocoma paradoxa* was used as an outgroup.

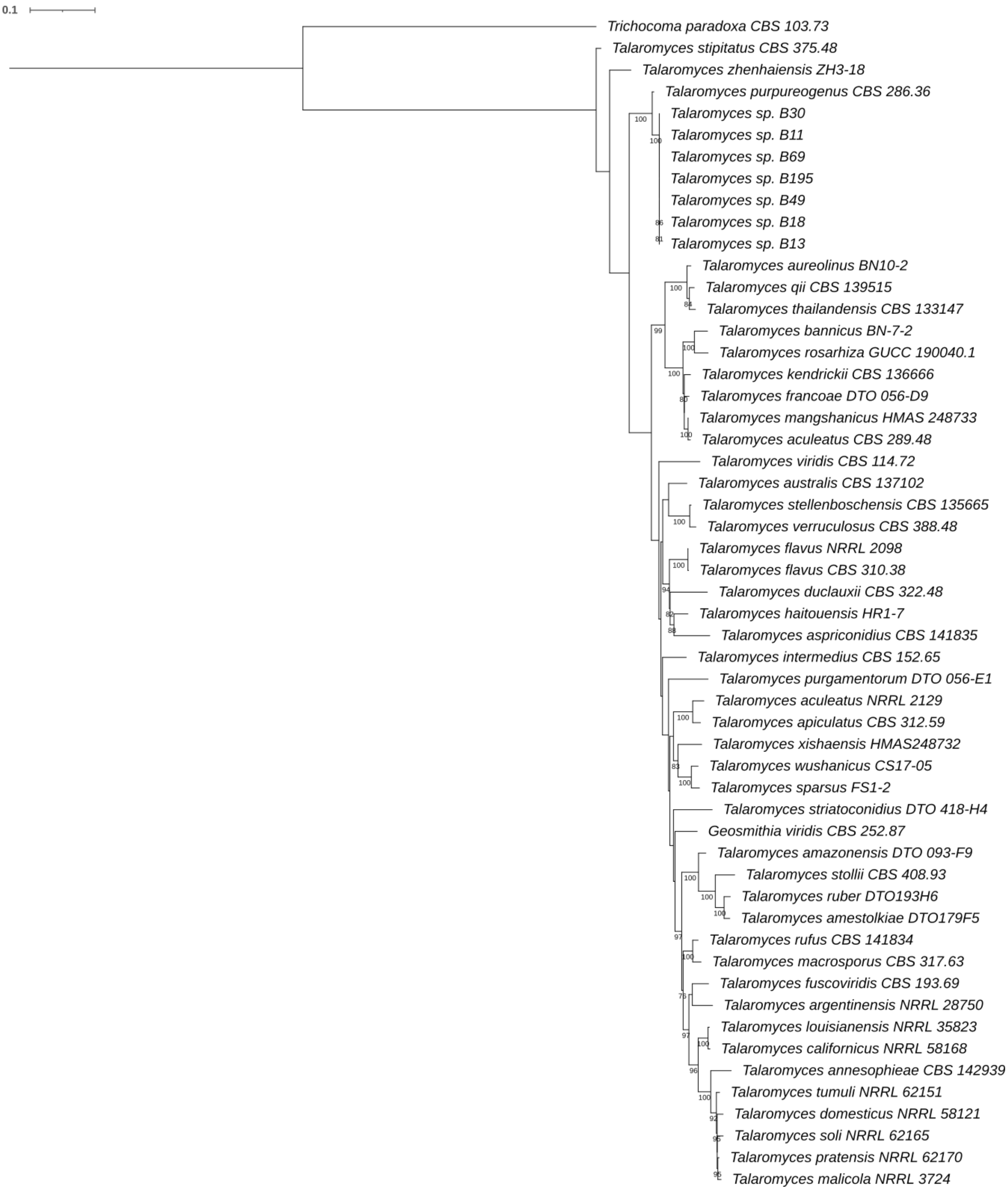

Figure S3: Maximum likelihood phylogenetic tree based on RBPII gene region. 54tax Support in the nodes is represented by bootstrap values >70. *Trichocoma paradoxa* was used as an outgroup.

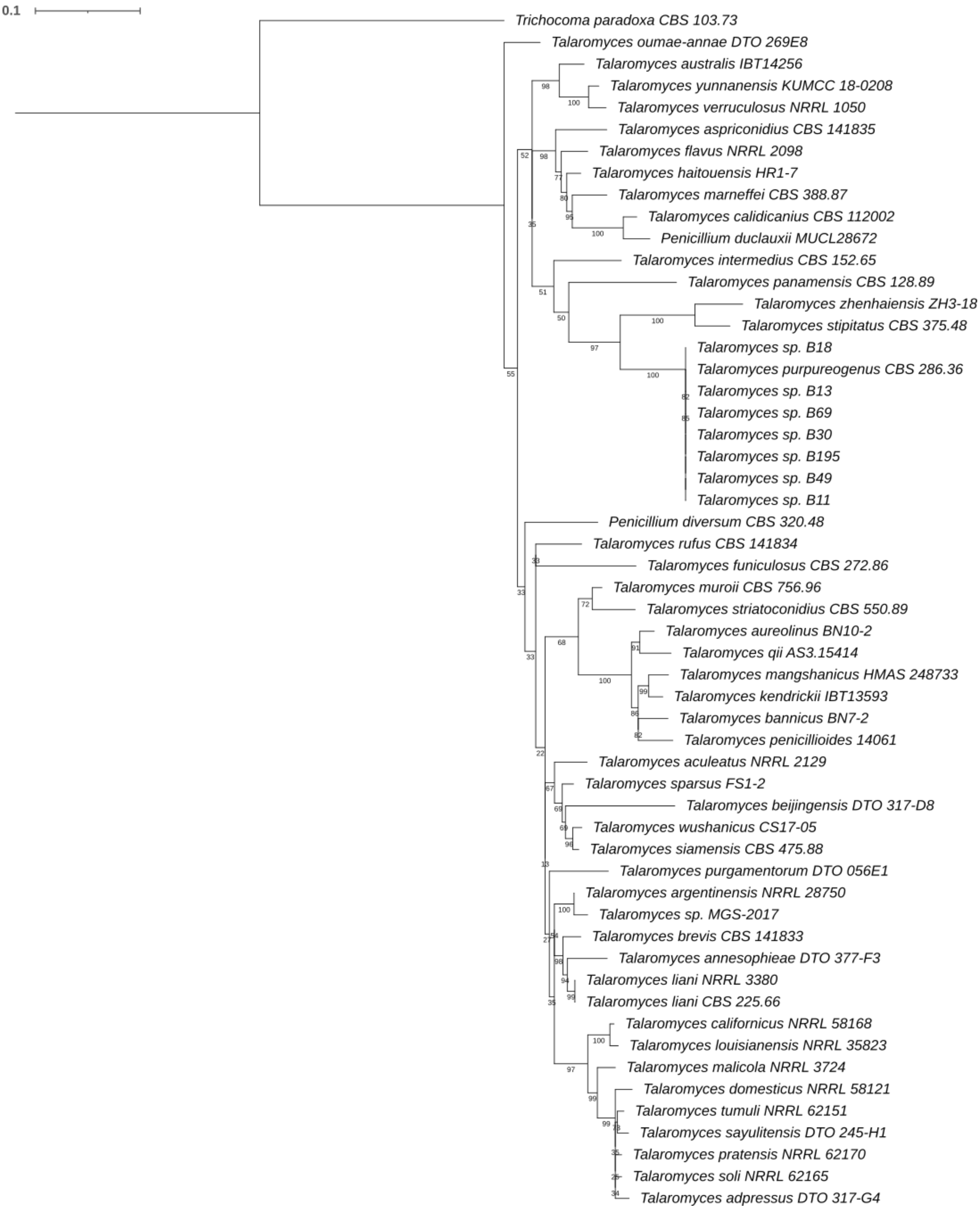

Figure S4: Maximum likelihood phylogenetic tree based on Ben2A gene region. 55tax Support in the nodes is represented by bootstrap values >70. *Trichocoma paradoxa* was used as an outgroup.

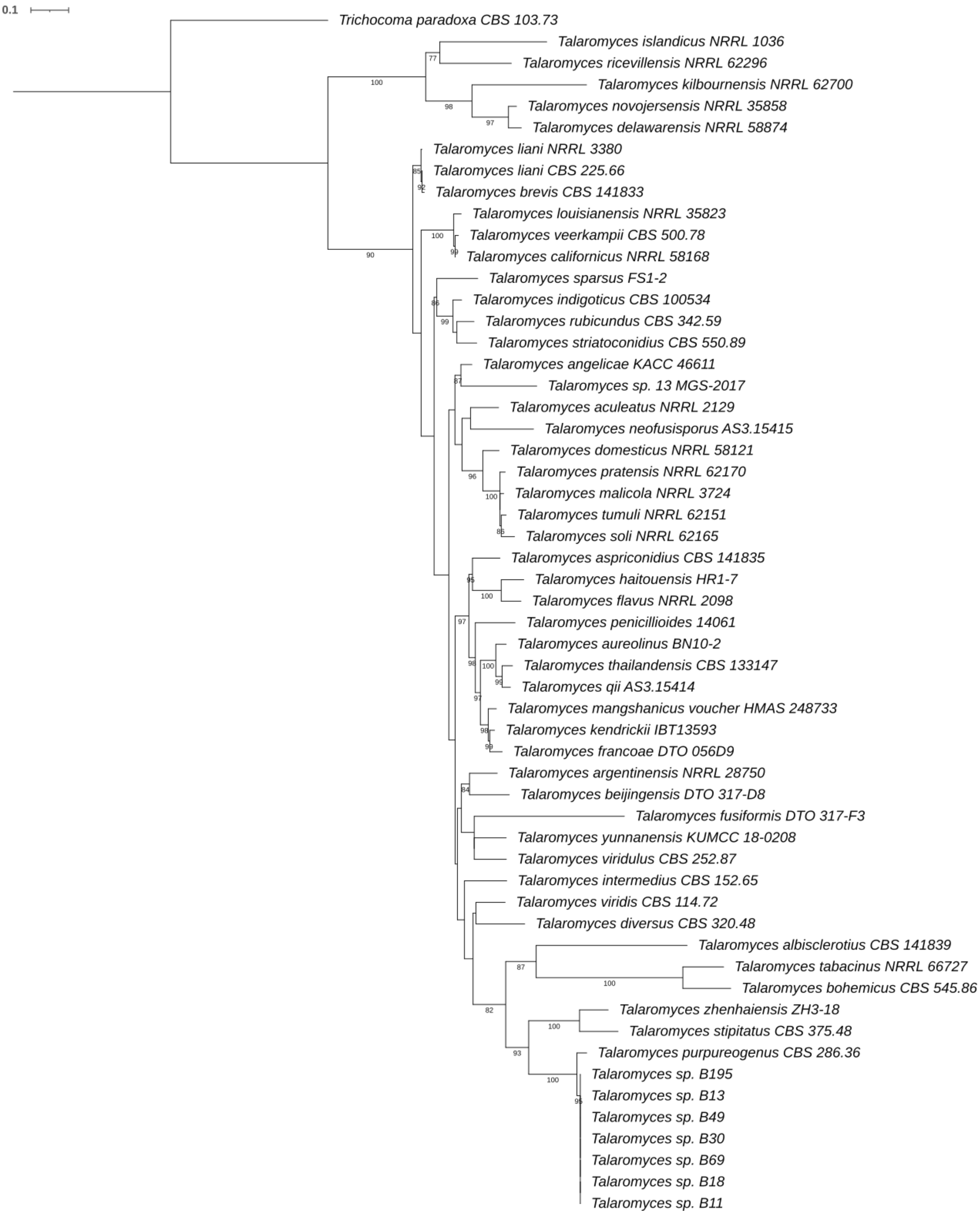

Figure S5: Maximum likelihood phylogenetic tree based on CaM gene region. 58tax Support in the nodes is represented by bootstrap values >70. *Trichocoma paradoxa* was used as an outgroup.

Kamenny Malikov, county Jindrichuv Hradec (49.2143497N, 15.1181131E), 526 m.a.s.l.

The apiaries are located in a small village, surrounded by forests, meadows and agricultural land and low level of urbanization within more than 6 km range. The anthropogenic pressure in this rural area is considered low. One of the apiaries (**KM agro**) is located at the canola field. Samples from six out of sixteen hives were collected in the first apiary – **KM Zirovnice** and from four out of ten in April and July 2019 and 2020 and in the second apiary – **KM agro** in April and July 2020.

CB campus, Ceske Budejovice (48.9773203N, 14.4548403E) 388 m.a.s.l.

The sampling spot is located in the urban area in the campus of the University of South Bohemia. The apiary is in the close vicinity (~ 2 km) to parks and small portion of agricultural land, roads and highway (within ~ 6-km range). Samples from all four hives were collected in April and July 2020.

CB Litvinovice, Ceske Budejovice (48.9658617N, 14.4404236E) 418 m.a.s.l.

This area is located in the suburb of Ceske Budejovice, close to CB campus (~ 1.6 km, see the characterization of CB campus). The hives are placed directly at the agricultural land. Samples from four out of ten hives were collected in April and July 2020.

CB Kroclov, Ceske Budejovice (48.9053261N, 14.3835472E), 540 m.a.s.l.

The apiary is located in an urban periphery surrounded by meadows, forests, agricultural land and low level of urbanization within ~ 6-km range. The anthropogenic pressure is considered low. Samples from all three hives were collected in April and July 2020.

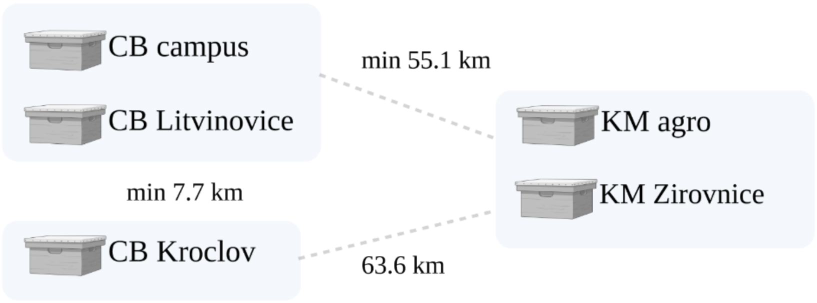

Figure S6: Approximate distances in straight line among the sampling spots in South Bohemia (Czech Republic). The apiaries in the light blue rectangles are within 1.5-2 km from each other. The values show the minimum distance (to the closer apiary in the area). Created with BioRender.com

Table S2: *Talaromyces* isolates from bee bread of *A. mellifera* collected from different locations in South Bohemia. The colonies were cultivated seven days at 28 °C on SDA.

Isolate	Sampling time<br>and location	Closest hit with Genbank<br>Identity [%]
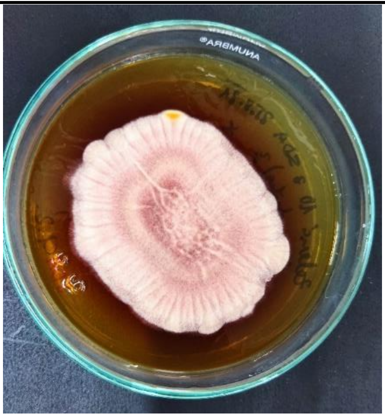	April 2020<br>CB campus	*T. purpureogenus*<br>99.27
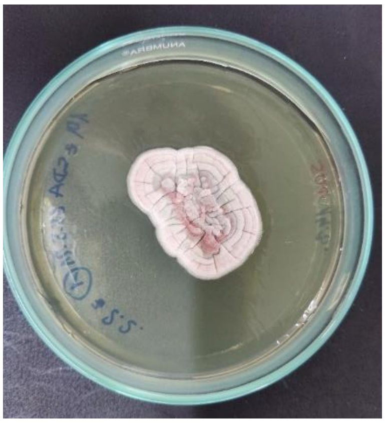	April 2020<br>CB campus	*T. purpureogenus*<br>97.94
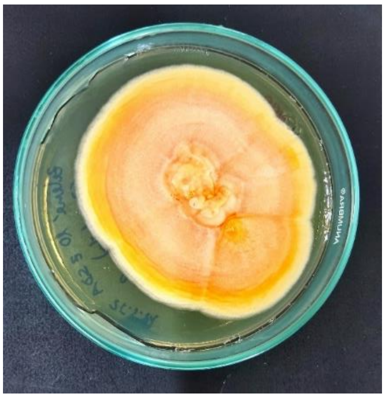	April 2020<br>KM Zirovnice	*T. purpureogenus*<br>99.38
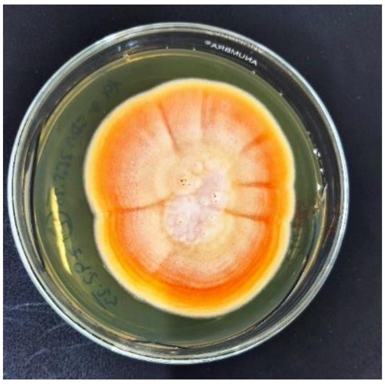	April 2020<br>KM Zirovnice	*T. purpureogenus*<br>98.92

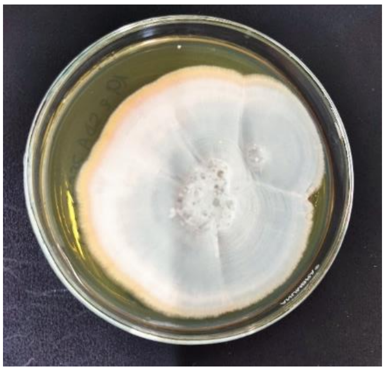	April 2020<br>KM Zirovnice	*T. purpureogenus*<br>99.38
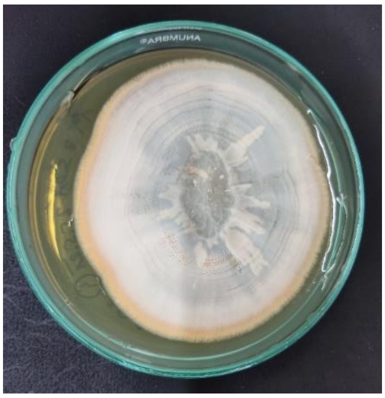	April 2020<br>CB Kroclov	*T. purpureogenus*<br>99.38
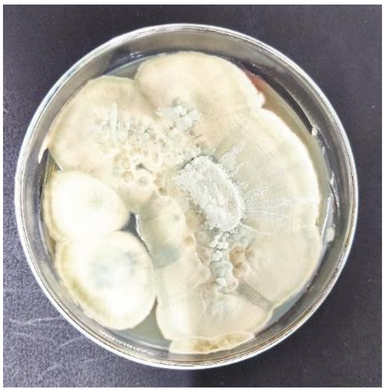	April 2020<br>CB Kroclov	*T. purpureogenus*<br>97.23
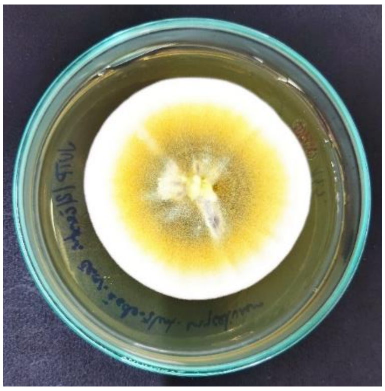	April 2020<br>CB campus	*T. piceae*<br>98.27

Table S3: Cosine similarities and grouping of the extracts on different media.

Order	Strain	Medium	Medium-type	Cosine	Metabolic group (0.8)
1	B69	MP	liquid	0.00	1
2	B11	MP	liquid	0.83	1
3	B49	MP	liquid	0.70	2
4	B30	MP	liquid	0.91	2
5	B13	MP	liquid	0.63	3
6	control	MP	liquid	0.49	4
7	control	MP	liquid	0.98	4
8	B195	MP	liquid	0.13	5
9	B18	MP	liquid	0.60	6
10	control	CYA	solid	0.15	7
11	control	YES	solid	0.93	7
12	B13	YES	solid	0.54	8
13	B13	CYA	solid	0.60	9
14	B11	YES	solid	0.10	10
15	B11	CYA	solid	0.88	10
16	B69	CYA	solid	0.02	11
17	B49	CYA	solid	0.93	11
18	B69	YES	solid	0.77	12
19	B49	YES	solid	0.60	13
20	B30	YES	solid	0.49	14
21	B30	CYA	solid	0.69	15
22	B195	YES	solid	0.36	16
23	B195	CYA	solid	0.48	17
24	B18	YES	solid	0.45	18
25	B18	CYA	solid	0.20	19

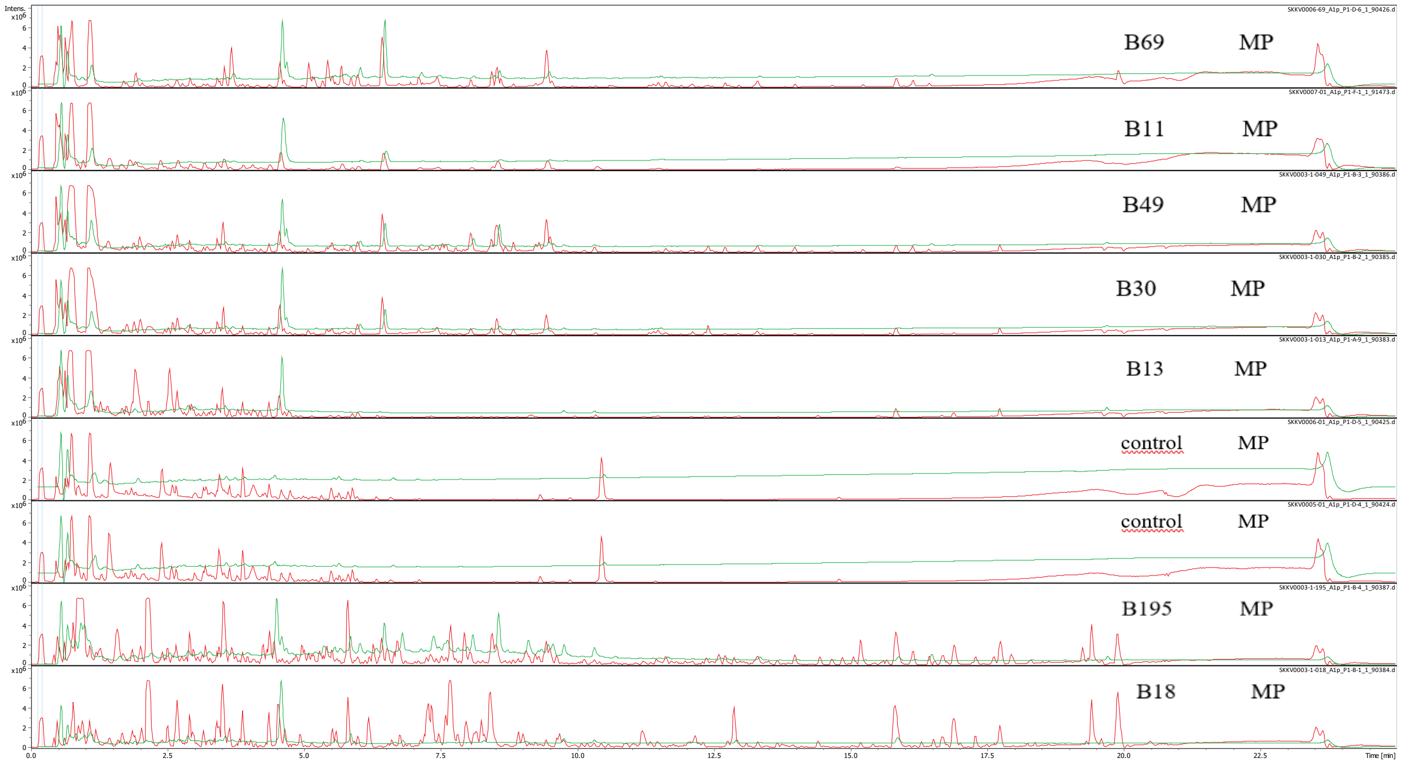

Figure S7: Chromatograms 1-9 in the clustering order (see table 3). BPC is depicted in red, UV absorption from 205-640 nm in green.

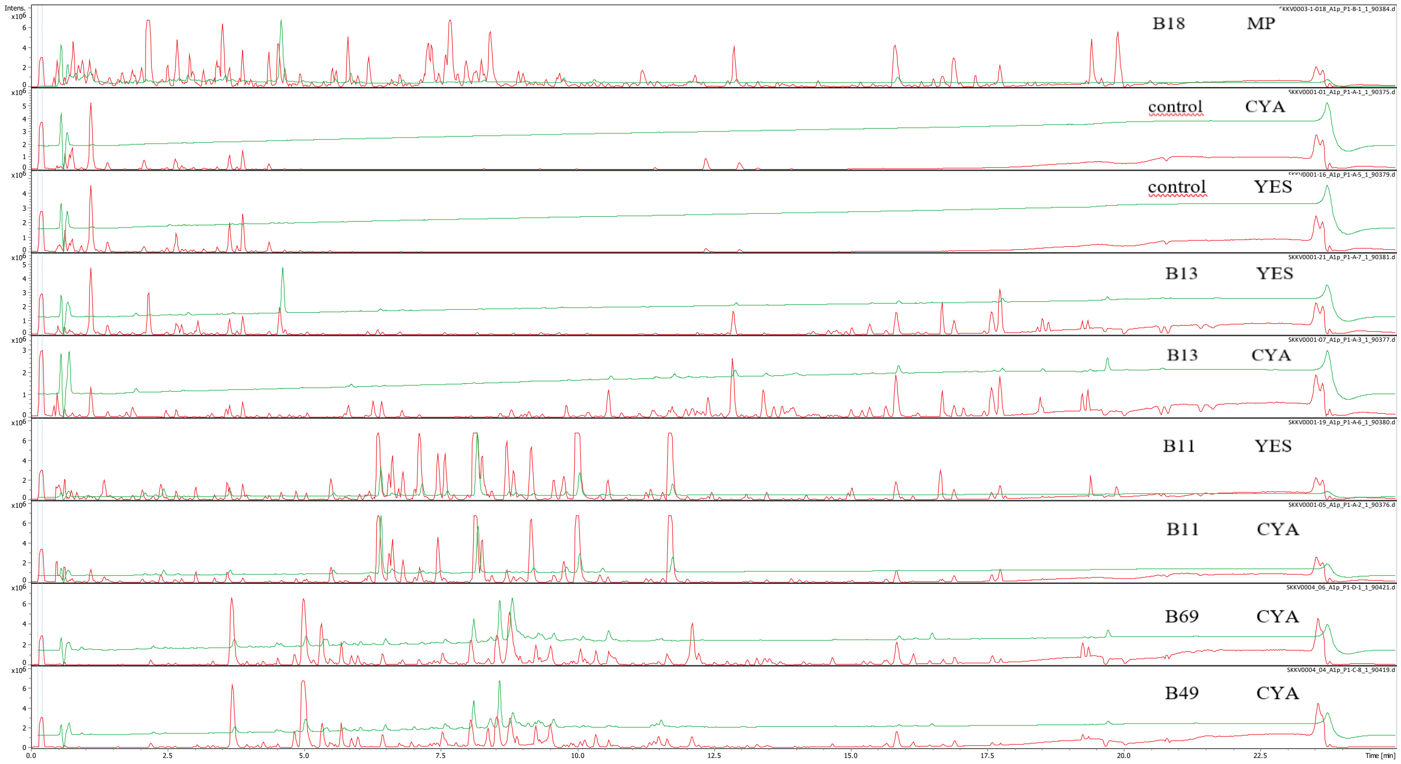

Figure S8: Chromatograms 9-17 in the clustering order (see table 3). BPC is depicted in red, UV absorption from 205-640 nm in green.

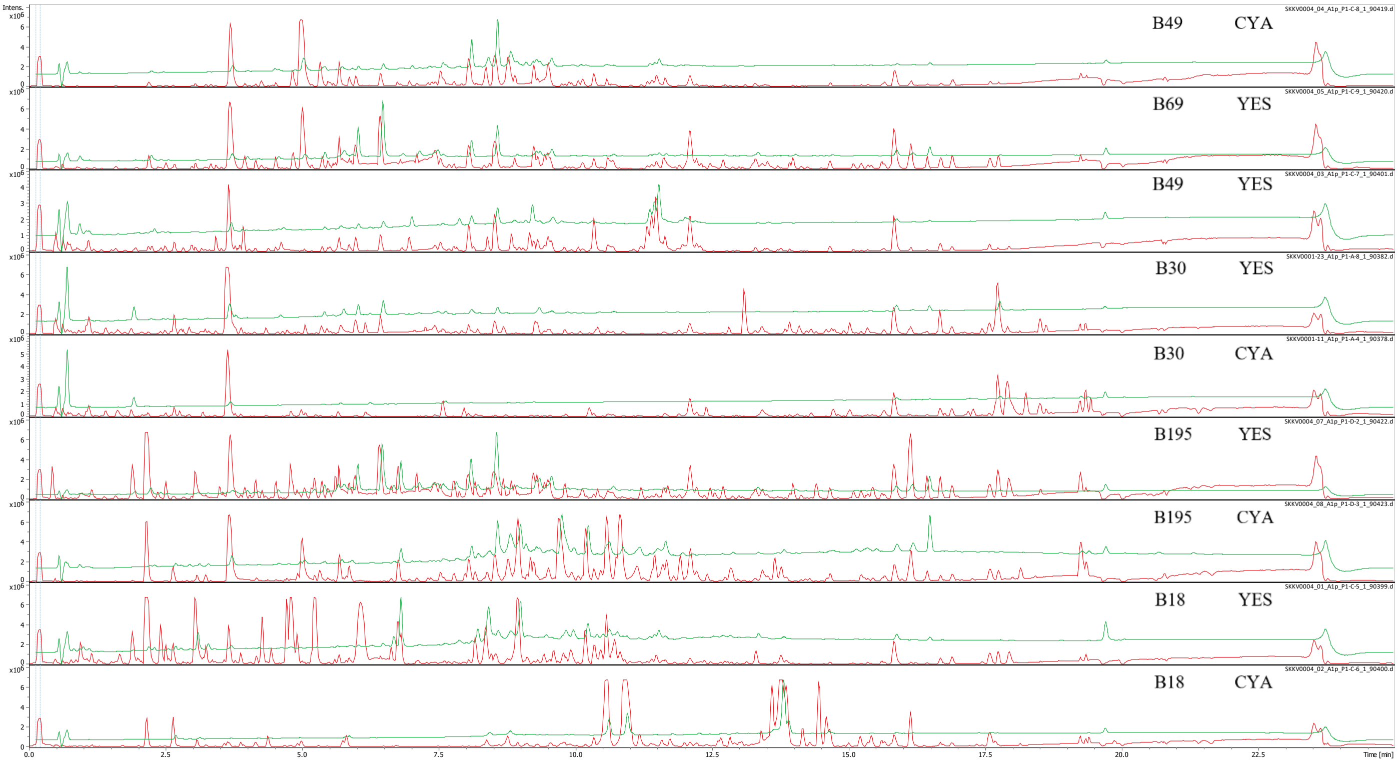

Figure S9: Chromatograms 17-25 in the clustering order (see table 3). BPC is depicted in red, UV absorption from 205-640 nm in green.

Table S4: Minimum inhibitory concentrations (MIC µg/mL) of the crude methanol extracts from *Talaromyces purpureogenus* strains

Strains	*B. subtilis* DSM10	*E. coli* ATCC35218	*P. lautus* DSM3035 FH1920	*P. lactis* FH1832	*Paenibacillus* sp. ST133196	*Paenibacillus* sp. ST514408
B11	> 2000	> 2000	> 2000	2000	> 2000	> 2000
B13	> 2000	> 2000	> 2000	> 2000	> 2000	> 2000
B18	> 2000	> 2000	500	500	> 2000	> 2000
B30	> 2000	> 2000	> 2000	2000	> 2000	> 2000
B49	> 2000	> 2000	> 2000	> 2000	> 2000	> 2000
B69	> 2000	> 2000	2000	> 2000	> 2000	> 2000
B195	2000	> 2000	2000	2000	> 2000	> 2000

Table S5: The minimum inhibitory concentration (MIC µg/mL) of the reference antibiotics evaluated by the microtiter turbidity test

Standards	MIC µg/mL					
Ciprofloxacin	0.06 - 0.03	0.008 - 0.002	0.125	0.125	0.25	0.5
Cefotaxim	0.25 - 0.125	0.06 - 0.03	2		4 - 2	0.25 - 0.125
Gentamicin	0.125 - 0.06	2 - 1	1 - 0.5	0.25	0.125	0.125

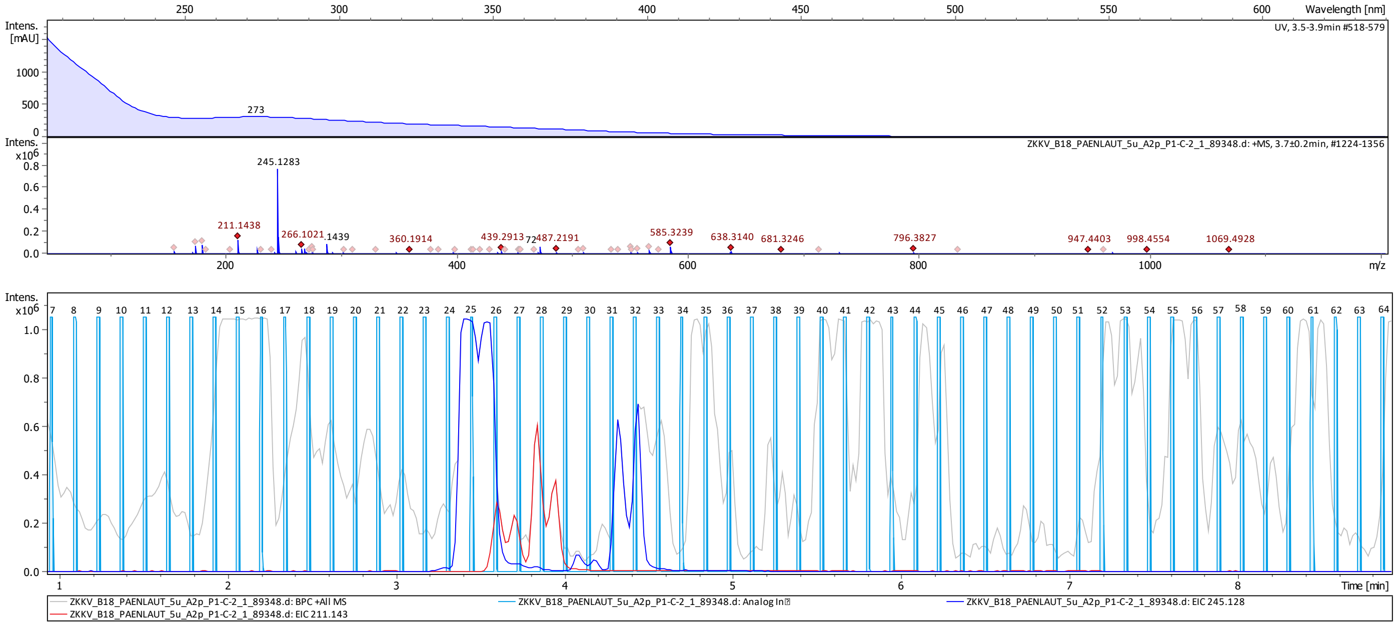

Figure S10: Analysis of the fractions 26-28 of the methanol extract from *Talaromyces purpureogenus* B18. Top: Average MS spectrum of fractions 26-28. The values *m/z* 245.1283 $[M+H]^+$ and *m/z* 211.1438 $[M+H]^+$ correspond to the molecular formulas $C_{14}H_{16}N_2O_2$ and $C_{11}H_{18}N_2O_2$, respectively. Bottom: Overlaid Base peak chromatogram (grey), fraction collector signals (blue bars) and extracted ion chromatogram of *m/z* 245.1283±0.005 $[M+H]^+$ (blue) and *m/z* 211.1438±0.005 $[M+H]^+$ (red).

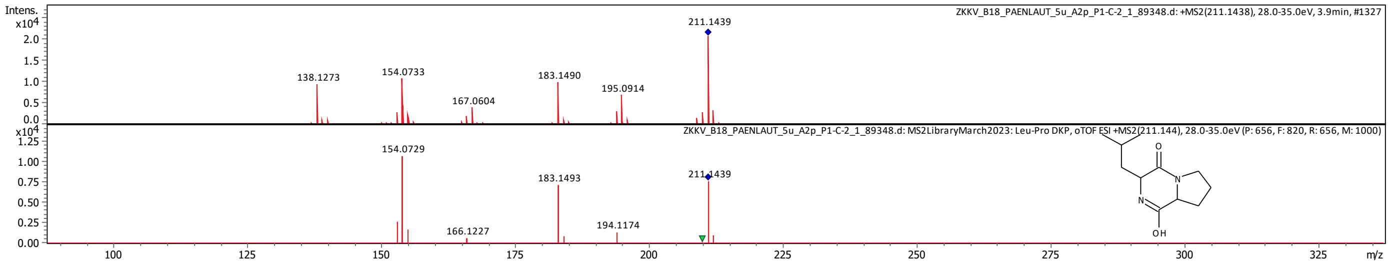

Figure S11: MS/MS spectrum of the precursor ion at m/z 211.1438 identified as cyclo-Leu-Pro from the internal MS/MS database.

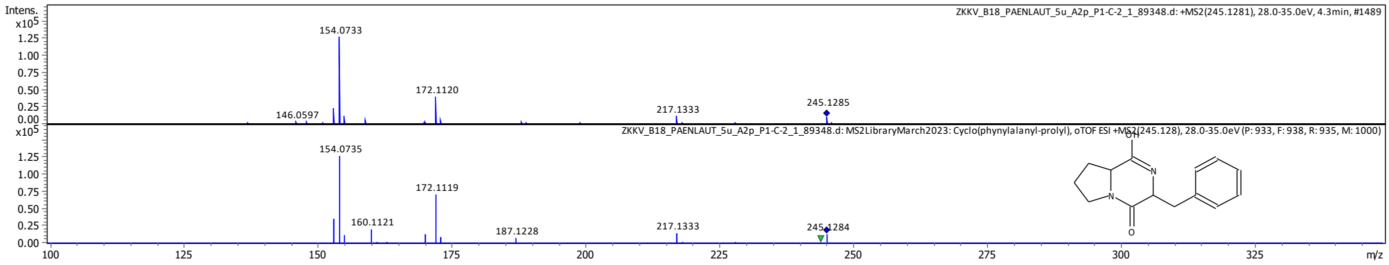

Figure S12: MS/MS spectrum of the precursor ion at *m/z* 245.1285 $[M+H]^+$ identified as cyclo-Phe-Pro from the internal MS/MS database.

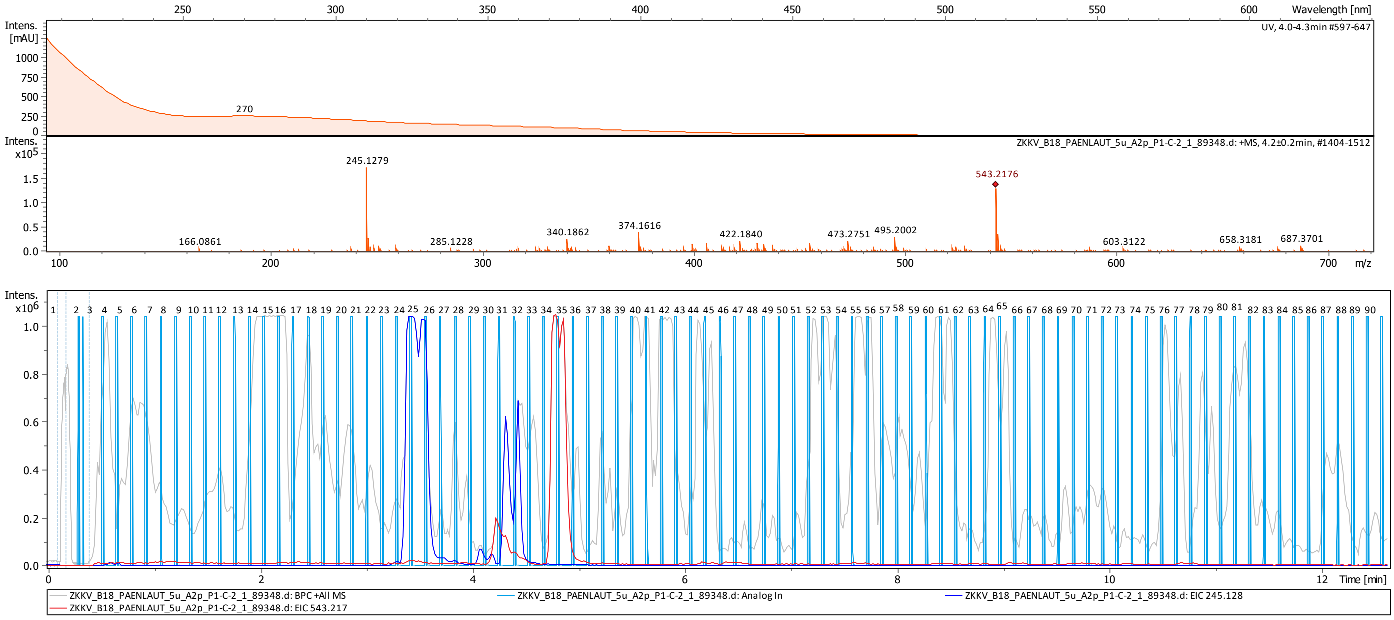

Figure S13: Analysis of the fractions 30-31 of the methanol extract from *Talaromyces purpureogenus* B18. Top: Average MS spectrum of fractions 30-31. The values *m/z* 245.1282 $[M+H]^+$ and *m/z* 543.2176 $[M+H]^+$ correspond to the molecular formulas $C_{14}H_{16}N_2O_2$ (isomers of cyclo-Phe-Pro) and $C_{24}H_{34}N_2O_{12}$, respectively. Bottom: Overlaid Base peak chromatogram (grey), fraction collector signals (blue bars) and extracted ion chromatogram of *m/z* 245.1283±0.005 $[M+H]^+$ (blue) and *m/z* 543.2176±0.005 $[M+H]^+$(red).

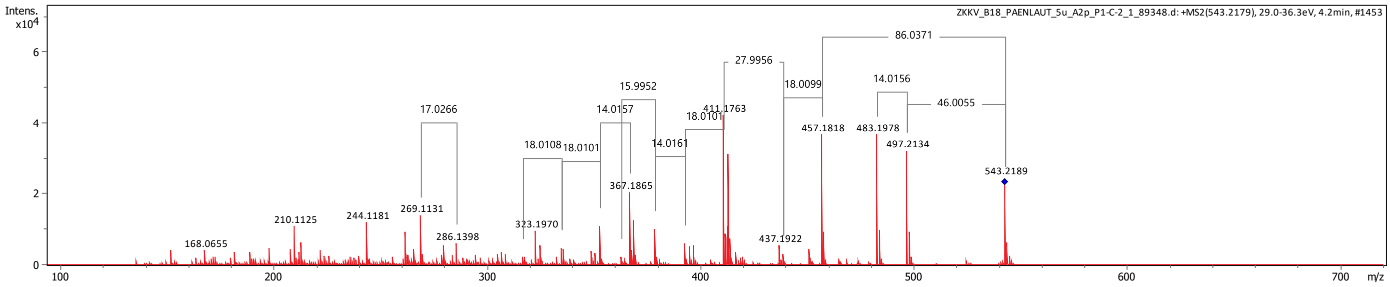

Figure S14: MS/MS spectrum of the precursor ion at *m/z* 543.2189 $[M+H]^+$, for which the molecular formula $C_{24}H_{34}N_2O_{12}$ was assigned. A molecular formula search in the commercial natural product databases retrieved no hits. Identity of the compound is unknown.

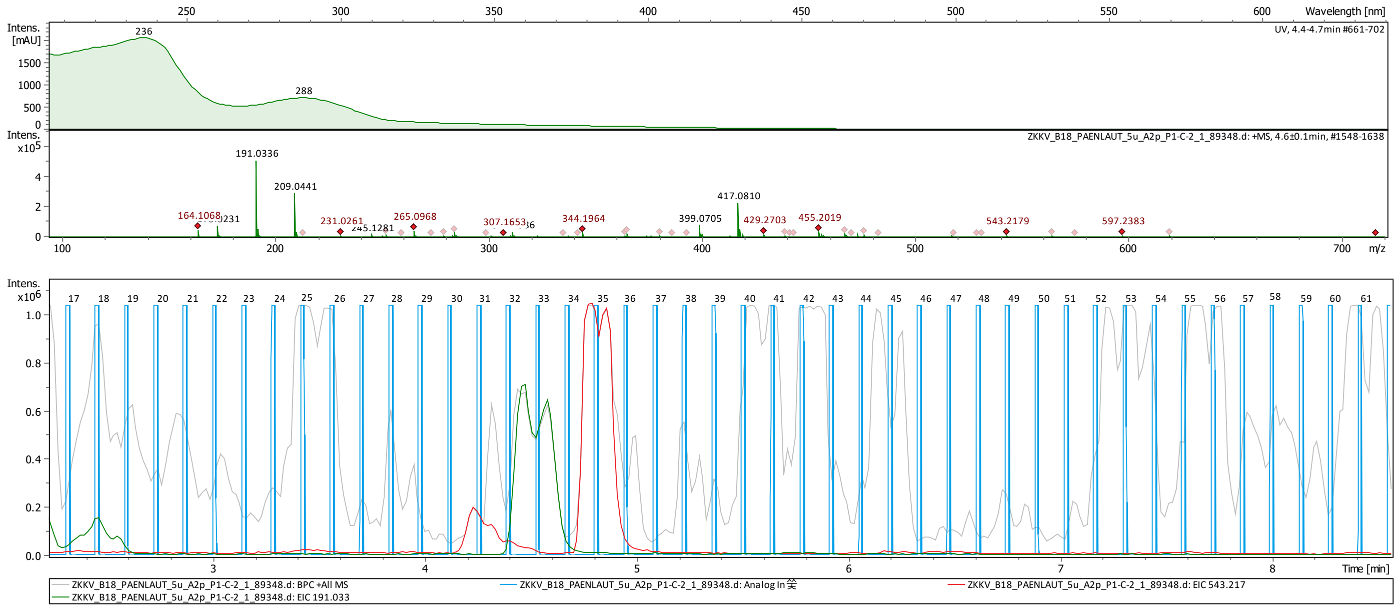

Figure S15: Analysis of the fractions 33-34 of the methanol extract from *Talaromyces purpureogenus* B18. Top: Average MS spectrum of fractions 33-34. The value *m/z* 543.2176 $[M+H]^+$ corresponds to the molecular formula $C_{24}H_{34}N_2O_{12}$, whereas the values *m/z* 191.0334 $[M-H_2O+H]^+$, *m/z* 209.0434 $[M+H]^+$ and *m/z* 417.0807 $[2M-2H_2O+H]^+$ correspond to $C_{10}H_8O_5$. Bottom: Overlaid Base peak chromatogram (grey), fraction collector signals (blue bars) and extracted ion chromatograms of *m/z* 543.2176±0.005 $[M+H]^+$ (red) and *m/z* 191.0334±0.005 $[M+H]^+$ (green).

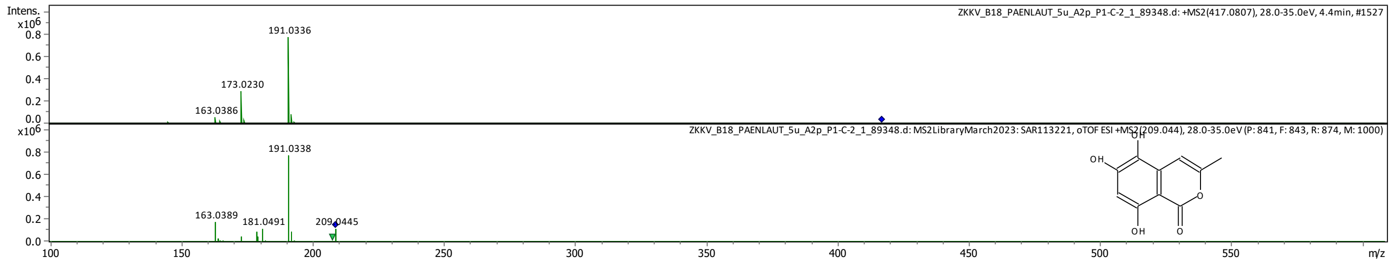

Figure S16: MS/MS spectrum of the precursor ion at *m/z* 417.0807 $[2M-H_2O+H]^+$). The assigned molecular formula $C_{10}H_8O_5$ was identified as 5,6,8-trihydroxy-3-methyl-2-benzopyran-1-one based on the internal MS/MS database.

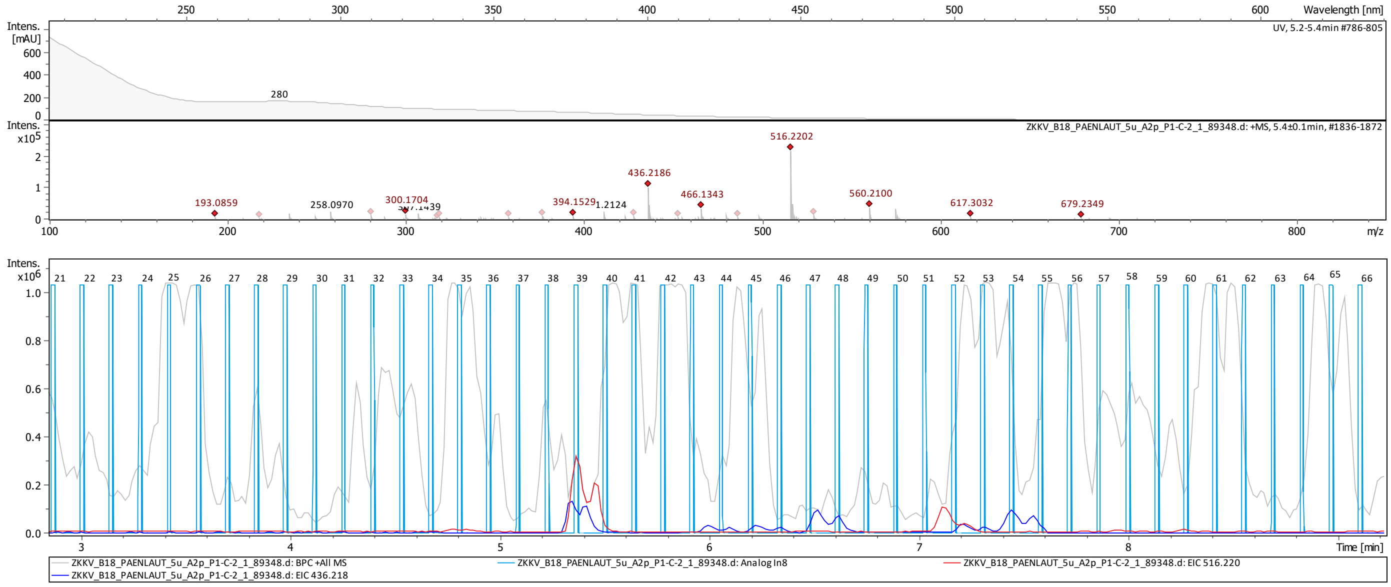

Figure S17: Analysis of the fractions 39 of the methanol extract from *Talaromyces purpureogenus* B18. Top:

Average MS spectrum of fraction 39. The value *m/z* 516.2204 $[M+H]^+$ corresponds to the molecular formula $C_{22}H_{33}N_3O_{11}$ and the values *m/z* 436.5186 $[M+2H]^{2+}$ and *m/z* 871.4282 $[M+H]^+$ to $C_{39}H_{62}N_6O_{16}$. Bottom: Overlaid Base peak chromatogram (grey), fraction collector signals (blue bars) and extracted ion chromatograms of *m/z* 516.2202±0.005 $[M+H]^+$ (red) and *m/z* 436.2186±0.005 $[M+H]^+$ (blue).

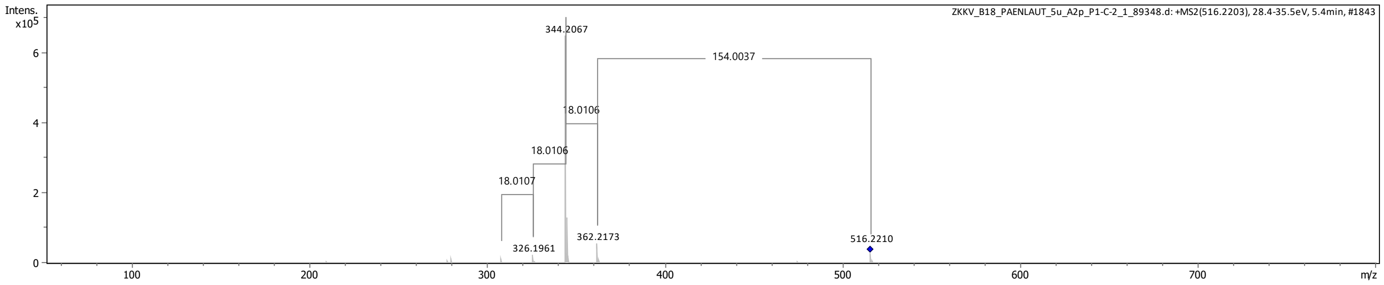

Figure S18: MS/MS spectrum of the precursor ion at *m/z* 516.2204 $[M+H]^+$, for which the molecular formula $C_{22}H_{33}N_3O_{11}$ was assigned. A molecular formula search in the commercial natural product databases retrieved no hits.

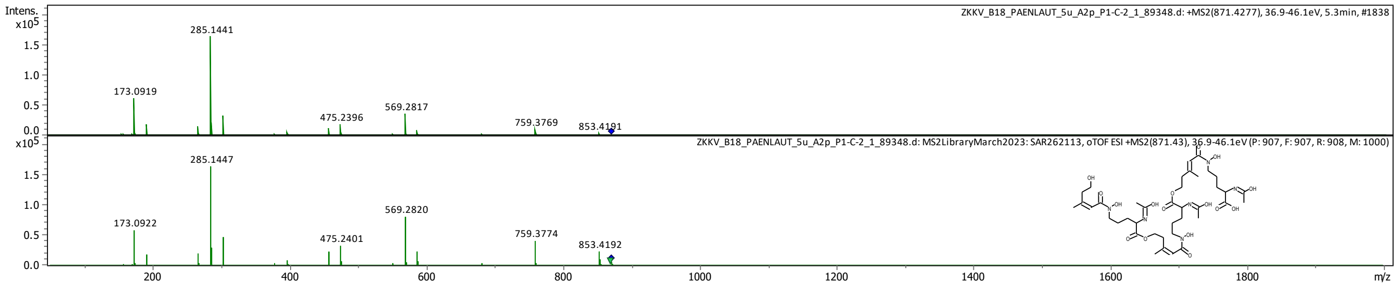

Figure S19: MS/MS spectrum of the precursor ion at *m/z* 871.4282 $[M+H]^+$ identified as L-Ornithine, N^2-acetyl-N^5-hydroxy-N^5-(5-hydroxy-3-methyl-1-oxo-2-pentenyl)-, trimol. ester, (*Z*,*Z*,*Z*)- (9CI):

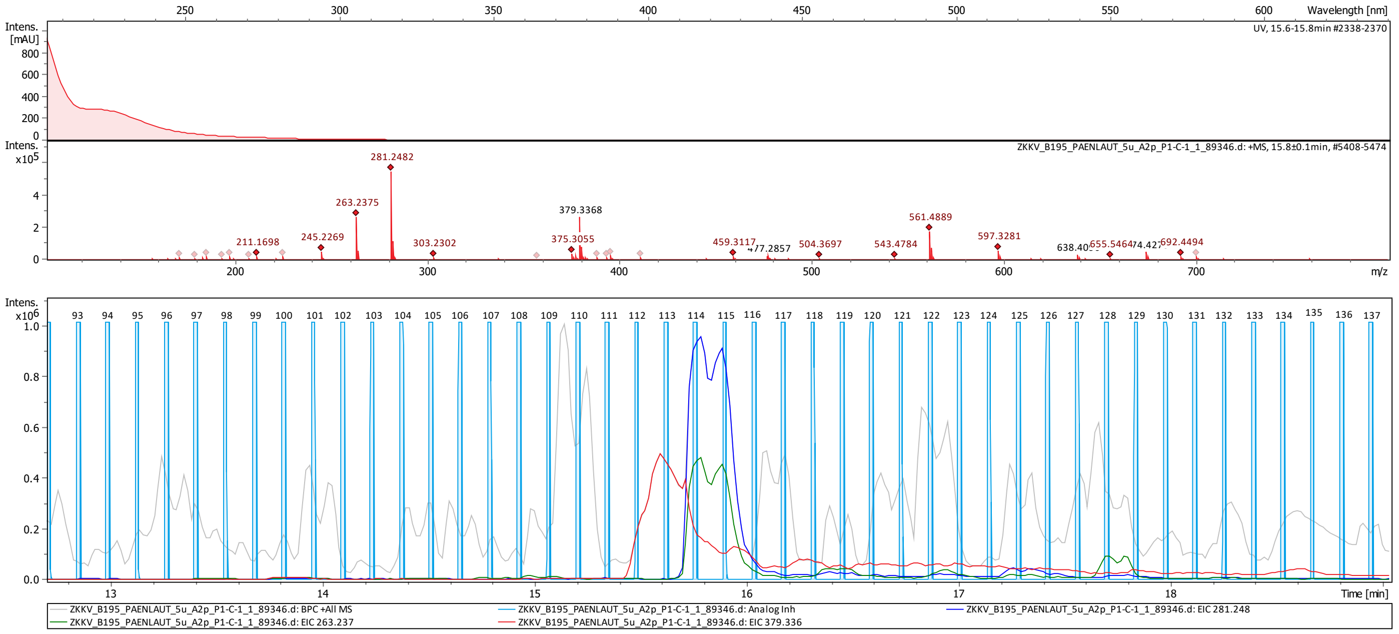

Figure S20: Analysis of the fraction 114 of the methanol extract from *Talaromyces purpureogenus* B18. Top: Average MS spectrum of fraction 114. The values *m/z* 281.2482 $[M+H]^+$ and *m/z* 379.3368 $[M+H]^+$ correspond to the molecular formulas $C_{18}H_{32}O_2$ and $C_{28}H_{42}$, respectively.

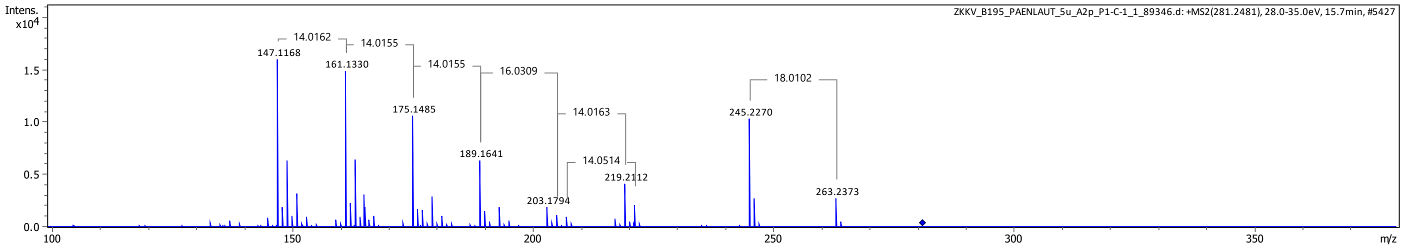

Figure S21: The MS/MS spectrum of the precursor ion at *m/z* 281.2482 $[M+H]^+$. The predicted molecular formula search of the major ion ($C_{18}H_{32}O_2$) retrieved 103 hits in DNP.
